# DV200 Index for Assessing RNA Integrity in Next-Generation Sequencing

**DOI:** 10.1155/2020/9349132

**Published:** 2020-02-25

**Authors:** Takehiro Matsubara, Junichi Soh, Mizuki Morita, Takahiro Uwabo, Shuta Tomida, Toshiyoshi Fujiwara, Susumu Kanazawa, Shinichi Toyooka, Akira Hirasawa

**Affiliations:** ^1^Okayama University Hospital Biobank, Okayama University Hospital, Japan; ^2^Department of Surgery, Division of Thoracic Surgery, Kindai University Faculty of Medicine, Japan; ^3^Department of General Thoracic Surgery, Breast and Endocrinological Surgery, Okayama University Graduate School of Medicine, Dentistry and Pharmaceutical Sciences, Japan; ^4^Department of Biomedical Informatics, Okayama University Graduate School of Interdisciplinary Science and Engineering in Health Systems, Japan; ^5^Department of Biobank, Okayama University Graduate School of Medicine, Dentistry and Pharmaceutical Sciences, Japan; ^6^Department of Gastroenterological Surgery, Okayama University Graduate School of Medicine, Dentistry and Pharmaceutical Sciences, Japan; ^7^Department of Radiology, Okayama University Graduate School of Medicine, Dentistry and Pharmaceutical Sciences, Japan; ^8^Department of Clinical Genomic Medicine, Okayama University Graduate School of Medicine, Dentistry and Pharmaceutical Sciences, Japan

## Abstract

Poor quality of biological samples will result in an inaccurate analysis of next-generation sequencing (NGS). Therefore, methods to accurately evaluate sample integrity are needed. Among methods for evaluating RNA quality, the RNA integrity number equivalent (RINe) is widely used, whereas the DV200, which evaluates the percentage of fragments of >200 nucleotides, is also used as a quality assessment standard. In this study, we compared the RINe and DV200 RNA quality indexes to determine the most suitable RNA index for the NGS analysis. Seventy-one RNA samples were extracted from formalin-fixed paraffin-embedded tissue samples (*n* = 30), fresh-frozen samples (*n* = 25), or cell lines (*n* = 16). After assessing RNA quality using the RINe and DV200, we prepared two kinds of stranded mRNA sequencing libraries. Finally, we calculated the correlation between each RNA quality index and the amount of library product (1^st^ PCR product per input RNA). The DV200 measure showed stronger correlation with the amount of library product than the RINe (*R*^2^ = 0.8208 for the DV200 versus 0.6927 for the RINe). Receiver operating characteristic curve analyses revealed that the DV200 was the better marker for predicting efficient library production than the RINe using a threshold of >10 ng/ng for the amount of the 1^st^ PCR product per input RNA (cutoff value for the RINe and DV200, 2.3 and 66.1%; area under the curve, 0.99 and 0.91; sensitivity, 82% and 92%; and specificity, 93% and 100%, respectively). Our results indicate that NGS libraries prepared using RNA samples with the DV200 value > 66.1% exhibit greater sensitivity and specificity than those prepared with the RINe values > 2.3. These findings suggest that the DV200 is superior to the RINe, especially for low-quality RNA, because it is a more consistent assessment of the amount of the 1^st^ NGS library product per input.

## 1. Introduction

Next-generation sequencing (NGS) has become an essential technology in molecular biology research and clinical assessment [1–3]. However, the quality of the input biological samples has a critical effect on NGS results. It is important to grasp the quality of NGS results before conducting NGS analyses in order to avoid wasting precious samples and to minimize cost and labor.

Several RNA quality indexes have been developed, including RNA integrity number equivalent (RINe) and DV200 metrics (percentage of RNA fragments > 200 nucleotides in size). RINe is generally and widely used for assessing RNA integrity, and it is based on a mathematical model that calculates an objective quantitative measurement of RNA degradation that represents the relative ratio of signal in the fast zone to the 18S peak signal.

The DV200 was developed by Agilent in 2014 as a tool to more accurately assess the quality of RNA samples (http://urx.red/OB4Y) and used as an RNA quality assessment standard even in the protocol published by Illumina. Values indicative of high quality can be obtained with the DV200 even for samples exhibiting weak 18S and 28S peaks if there is a sufficient volume of RNA fragments greater than 200 nt in length. However, the best practice for evaluating RNA quality remains uncertain. In this study, we compared the two RNA quality indexes in terms of the amount of the 1^st^ PCR product as preparation for NGS analyses in order to determine a much more suitable RNA quality index.

## 2. Materials and Methods

### 2.1. Data Collection

Seventy-one specimens were obtained at four sections of Okayama University Hospital (Center for Clinical Oncology; Department of Hematology, Oncology and Respiratory Medicine; Department of Respiratory Medicine; and Department of Thoracic, Breast and Endocrinological Surgery) during their own studies. All study protocols were approved by the Institutional Review Board/Ethical Committee of Okayama University, Okayama, Japan (reference numbers K1603-066, K1512-024, K1505-033, K1605-022, and K1808-009), and all participants signed written informed consent. Each section consigned an analysis by NGS to our biobank for its own research purpose and provided collected samples to Okayama University Hospital Biobank. This study uses only the data obtained in the steps of RNA extraction from the provided collected samples, preparation of the NGS library, and the NGS analysis, which were conducted at Okayama University Hospital Biobank. Detailed information regarding each of the samples used in the study is shown in Supplemental [Supplementary-material supplementary-material-1].

### 2.2. RNA Extraction and Quality Evaluation

RNA was extracted from frozen samples (*n* = 25) and cell lines (*n* = 16) using the RNeasy Mini kit (Qiagen, Hilden, Germany) or from formalin-fixed paraffin-embedded (FFPE) tissue samples (*n* = 30) using the RNeasy FFPE kit. RINe values were automatically determined on the basis of electropherograms generated using TapeStation HS RNA ScreenTape (Agilent Technologies, Santa Clara, CA, USA). We calculated the DV200 values on the basis of the same electropherograms using TapeStation Analysis software.

### 2.3. NGS Library Construction

NGS libraries were prepared using TruSeq RNA Access (Illumina, San Diego, CA, USA) (*n* = 63) or TruSight RNA Pan-Cancer (Illumina, San Diego, CA, USA) (*n* = 8). The NGS library preparation kits utilized the same workflows: fragmentation, cDNA synthesis, 1^st^ PCR, hybridization, 2^nd^ PCR, and cleanup, although hybridization probes were different. The amount of the NGS library product was quantified using a Qubit 2.0 fluorometer (Thermo Fisher, Waltham, MA, USA).

### 2.4. Receiver Operating Characteristic Curve Analysis

We generated receiver operating characteristic (ROC) curves using JMP 9.0.2 software (SAS Institute Japan, Osaka, Japan). We determined >10 ng/ng for the 1^st^ PCR product per input RNA as the threshold on the basis of the following factors: (1) 200 ng of 1^st^ PCR product is needed to proceed to the 2^nd^ PCR step for the NGS library preparation and (2) the minimum recommended input volume of RNA is 20 ng, as determined according to the following formula: 200 ng 1^st^ PCR product/20 ng input volume = 10 ng/ng.

## 3. Results

### 3.1. Distribution of RINe and DV200 Values

**Figure 1:**
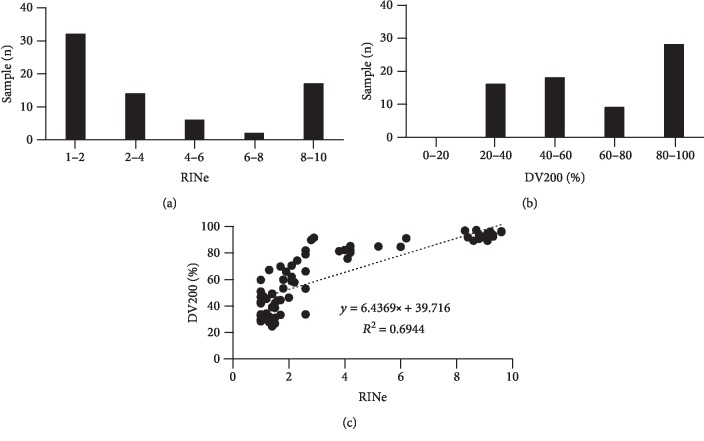
Relationship between RINe and DV200 values. (a, b) Distribution of RINe and DV200 values. Graphs show the distribution of RINe and DV200 values categorized in 2-point and 20-point increments, respectively. (c) Correlation between RINe and DV200 values. RINe and DV200 values were determined using TapeStation 2200.

The median values (range) were 2.1 (1.0–9.6) for the RINe and 66.1% (24.6–97.3%) for the DV200. The DV200 values were relatively scattered, whereas the RINe values exhibited two peaks at approximately 1 to 4 and 8 to 10 (Figures 1(a) and 1(b); Supplementary [Supplementary-material supplementary-material-1]).

### 3.2. Correlation between DV200 and RINe

As shown in Figure 1(c), the RINe and DV200 values were correlated (*R*^2^ = 0.6944). It should be noted that 12 of 32 (37.5%) samples with a low RINe value (<5) exhibited a high DV200 value (>70%), suggesting that the DV200, compared with RINe, has the potential to increase the number of samples available for the following assays.

### 3.3. RINe and DV200 Values and NGS Library Preparation

**Figure 2:**
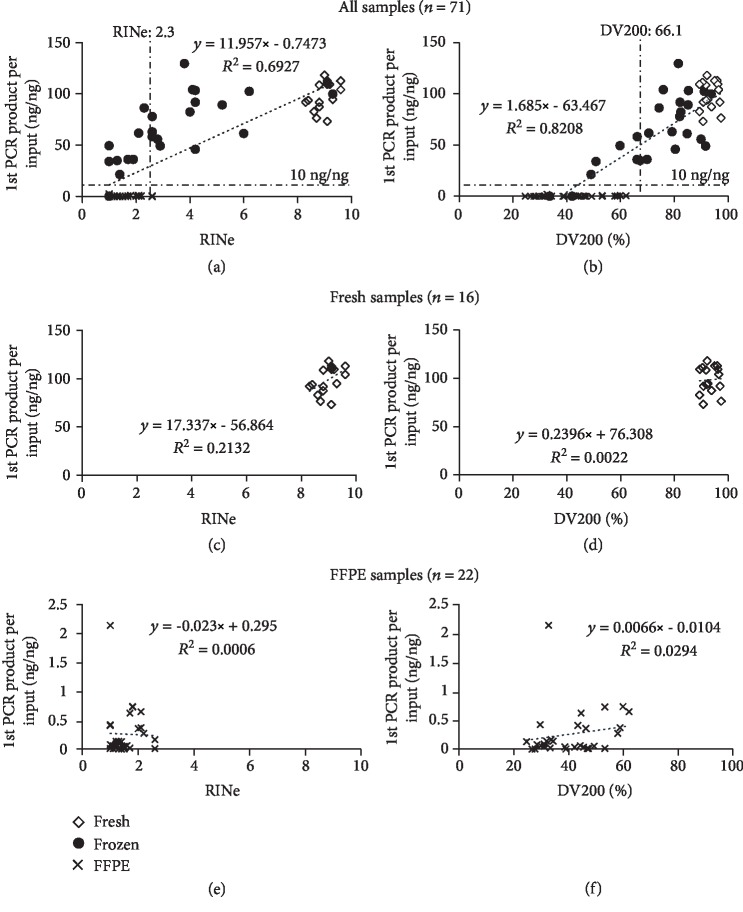
Correlation between RNA quality indexes and NGS library yields. (a, b) Correlation between RINe and DV200 of all samples in terms of amount of the 1^st^ NGS library product per input (◇: fresh, ●: frozen, and ×: FFPE). NGS libraries were prepared using TruSeq RNA Access or TruSight RNA Pan-Cancer. The amount of the 1^st^ NGS library product was measured using Qubit. Threshold lines are drawn for the amount of the 1^st^ NGS library product (10 ng/ng) and the cutoff value (RINe: 2.3 and DV200: 66.1) on the basis of receiver operating characteristic (ROC) curve analysis. (c)–(f) are segregated from all samples depending on sample type: fresh sample (◇: c, d) or FFPE sample (×: e, f).

The median of the 1^st^ NGS library product per input was 41.0 ng/*μ*l (0.01–129.5 ng/*μl*) (Supplemental [Supplementary-material supplementary-material-1]). Both the RINe and DV200 values correlated positively with the amount of the 1^st^ NGS library product, although the DV200 exhibited a better correlation than the RINe index (*R*^2^ = 0.8208 versus 0.6927, respectively) (Figures 2(a) and 2(b)). The fresh and FFPE samples were extracted and analyzed separately from the other samples to investigate the effects of different sample types. In the fresh samples, a high RINe value, a high DV200 value, and a sufficient amount of the 1^st^ NGS library product were obtained (more than 8.3, 89.32, and 73.15 ng/ng, respectively), even though the *R*^2^ value of the RINe was higher than that of the DV200 (Figures 2(c) and 2(d)). Although the amount of the 1^st^ NGS library product was low in all FFPE samples, the DV200 showed better *R*^2^ value than the RINe (0.0294 versus 0.0006), indicating that the DV200 is useful for evaluating RNA in low-quality samples such as FFPE.

### 3.4. Receiver Operating Characteristic Curve Analyses

**Figure 3:**
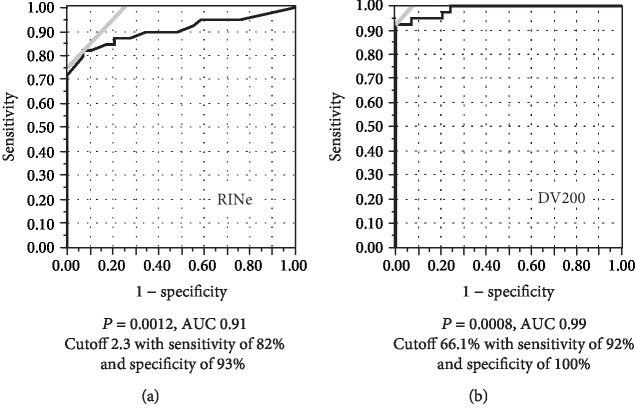
Receiver operating characteristic curves for RINe and DV200. ROC curves for the RINe and DV200 indexes indicating the most efficient amount (more than 10 ng/ng) of the 1^st^ PCR library product per input. The area under the curve for DV200 was greater than that for the RINe index (0.99 with *P* = 0.0008 and 0.91 with *P* = 0.0012, respectively).

The analysis of ROC curves indicated that the optimal RINe and DV200 cutoff values were 2.3 and 66.1%, respectively, when >10 ng/ng for the 1st PCR product per RNA input was considered a sufficient amount for NGS. The area under the curve (AUC) for the DV200 was 0.99, with sensitivity of 92% and specificity of 100%, whereas the AUC for the RINe was 0.91, with sensitivity of 82% and specificity of 93% (Figures 3(a) and 3(b)).

## 4. Discussion

Remarkable progress in development of NGS technologies has made it possible to analyze a variety of specimens, including highly degraded materials such as 10-year-old FFPE samples [4]. The RINe has been widely used as an indicator of RNA quality in NGS, microarray, and qPCR [5–7]. However, the DV200 is more suitable than the RINe for quantification of RNA because it can be applied to evaluate not only RNAs extracted from fresh or frozen samples but also samples with lower RINe values, such as RNAs extracted from FFPE samples [8, 9]. In our study, the DV200 showed better correlation with the amount of the 1^st^ NGS library product compared with the RINe even for low-quality samples such as FFPE. Recently, paraffin-embedded RNA metrics (PERM) is also proposed as a novel indicator that is based on the intensity of fluorescence at specific time points using the Agilent 2100 Bioanalyzer (Agilent Technologies, Palo Alto, CA) [10]. Although we attempted to perform a PERM analysis, unfortunately, the TapeStation used in this study did not support the PERM analysis.

Furthermore, our study also revealed that the DV200 with a cutoff value of 66.1% provided greater AUC, sensitivity, and specificity than the RINe (cutoff value 2.3) on the basis of the analysis of ROC curves. These results indicate that the DV200 with a cutoff value of 66.1% is more useful than the RINe for predicting whether a sufficient amount of high-quality 1^st^ NGS product can be obtained.

In addition to the 1^st^ NGS library product per input, we examined the effect of RNA quantification on quality metrics of RNA sequencing (RNA-seq): duplicates, reads not mapped, and nonspecific matches. As shown in Supplemental [Supplementary-material supplementary-material-1], the DV200 showed better *R*^2^ values than the RINe. Consistent with our report, another study reported a positive correlation between the DV200 value and the number of uniquely mapped NGS reads, which are reads mapped to one region of the reference genome [11]. By contrast, sample selection based on the RINe values reportedly provides no advantage for determining the quality of NGS reads [12]. In order to analyze some functional relationships between the RNA quality and the result of RNA-seq, we analyzed the transcripts per million (TPM) of protein coding genes (Supplemental [Supplementary-material supplementary-material-1]), and we found that the total TPM of protein coding genes in all the fresh samples (RINe > 8.0 and DV200 > 89%) exceeds 950,000 (meaning 95% of total RNA-seq reads). This result suggests that RNA-seq with high-quality input RNA using TruSeq RNA Access library preparation protocols could capture the whole picture of gene expression of protein coding genes with the least information loss. On the other hand, the total TPM of protein coding genes in all the FFPE samples (RINe < 3.0 and DV200 < 55%) ranged from 675,000 to 778,000 (meaning 67.5–77.8% of total RNA-seq reads) with one outlier (578,000). This result suggests that RNA-seq for low-quality input RNA may lead to the gene expression profiles with some information loss due to the potential RNA degradation/fragmentation. The total TPM of protein coding genes in frozen samples ranged widely from 150,000 to 970,000 on the basis of their RNA quality. On the other hand, in samples with RINe values of 2 or less, some samples had TPM values of more than 800,000, but others had TPM values of 800,000 or less. This result suggests that careful interpretation is required when using RNA with an RINe value of 2 or less.

These data suggest that the DV200 index is superior to RINe for assessing RNA integrity in order to obtain NGS results worthy of evaluation.

In general, the time required for tissue acquisition, fixation, and preservation is important for RNA quality [13, 14]. However, unfortunately, we could not obtain detailed information including ischemia time, interval from sample collection to formalin fixation, and formalin fixation duration. Currently, we are planning to obtain the duration of processing for sample preservation to investigate the effect of the duration of the preservation process on RNA quality as well as NGS libraries.

## 5. Conclusion

The DV200 index is a more consistent assessment of the amount of the 1^st^ NGS library product per input than the RINe index, especially for low-quality RNA. Therefore, we conclude that the DV200 is a beneficial RNA quality index for NGS analyses using degraded RNA samples such as those extracted from FFPE samples.

## Data Availability

The data used to support the findings of this study are available from the corresponding author upon request.

## Supplementary Materials

Supplementary 1 Supplemental Table 1. list of samples.

Supplementary 2 Supplemental Figure 1: correlation between RNA quality indexes and RNA-seq quality data. Supplemental Figure 2: functional relationship between the RNA quality and the result of RNA-seq.

## References

[1] Zhang Y. C., Zhou Q., Wu Y. L. (2017). The emerging roles of NGS-based liquid biopsy in non-small cell lung cancer. *Journal of Hematology & Oncology*.

[2] Fernandez-Marmiesse A., Gouveia S., Couce M. L. (2018). NGS technologies as a turning point in rare disease research, diagnosis and treatment. *Current Medicinal Chemistry*.

[3] Aly S. M., Sabri D. M. (2015). Next generation sequencing (NGS): a golden tool in forensic toolkit. *Archiwum Medycyny Sa̧dowej i Kryminologii*.

[4] Huang W., Goldfischer M., Babayeva S. (2015). Identification of a novel PARP14-TFE3 gene fusion from 10-year-old FFPE tissue by RNA-seq. *Genes, Chromosomes & Cancer*.

[5] Gibbons D. L., Lin W., Creighton C. J. (2009). Contextual extracellular cues promote tumor cell EMT and metastasis by regulating miR-200 family expression. *Genes & Development*.

[6] Sanz E., Yang L., Su T., Morris D. R., McKnight G. S., Amieux P. S. (2009). Cell-type-specific isolation of ribosome-associated mRNA from complex tissues. *Proceedings of the National Academy of Sciences of the United States of America*.

[7] Endrullat C., Glokler J., Franke P., Frohme M. (2016). Standardization and quality management in next-generation sequencing. *Applied & Translational Genomics*.

[8] Sanchez I., Betsou F., Mathieson W. (2019). Does vacuum centrifugal concentration reduce yield or quality of nucleic acids extracted from FFPE biospecimens?. *Analytical Biochemistry*.

[9] Landolt L., Marti H. P., Beisland C., Flatberg A., Eikrem O. S. (2016). RNA extraction for RNA sequencing of archival renal tissues. *Scandinavian Journal of Clinical and Laboratory Investigation*.

[10] Chung J. Y., Cho H., Hewitt S. M. (2016). The paraffin-embedded RNA metric (PERM) for RNA isolated from formalin-fixed, paraffin-embedded tissue. *BioTechniques*.

[11] Santoro S., Lopez I. D., Lombardi R. (2018). Laser capture microdissection for transcriptomic profiles in human skin biopsies. *BMC Molecular Biology*.

[12] Gallego Romero I., Pai A. A., Tung J., Gilad Y. (2014). RNA-seq: impact of RNA degradation on transcript quantification. *BMC Biology*.

[13] Sun H., Sun R., Hao M. (2016). Effect of duration of ex vivo ischemia time and storage period on RNA quality in biobanked human renal cell carcinoma tissue. *Annals of Surgical Oncology*.

[14] Srinivasan M., Sedmak D., Jewell S. (2002). Effect of fixatives and tissue processing on the content and integrity of nucleic acids. *The American Journal of Pathology*.

