# Supplementary material for: DV200 Index for Assessing RNA Integrity in Next-Generation Sequencing
Source: Biomed Res Int. 2020 Feb 25;2020:9349132. doi: 10.1155/2020/9349132 (PMC7063185; doi:10.1155/2020/9349132)
Supplement: Supplementary 2 — Supplemental Figure 1: correlation between RNA quality indexes and RNA-seq quality data. Supplemental Figure 2: functional relationship between the RNA quality and the result of RNA-seq. [file 9349132.f2.pptx]

## Slide 1
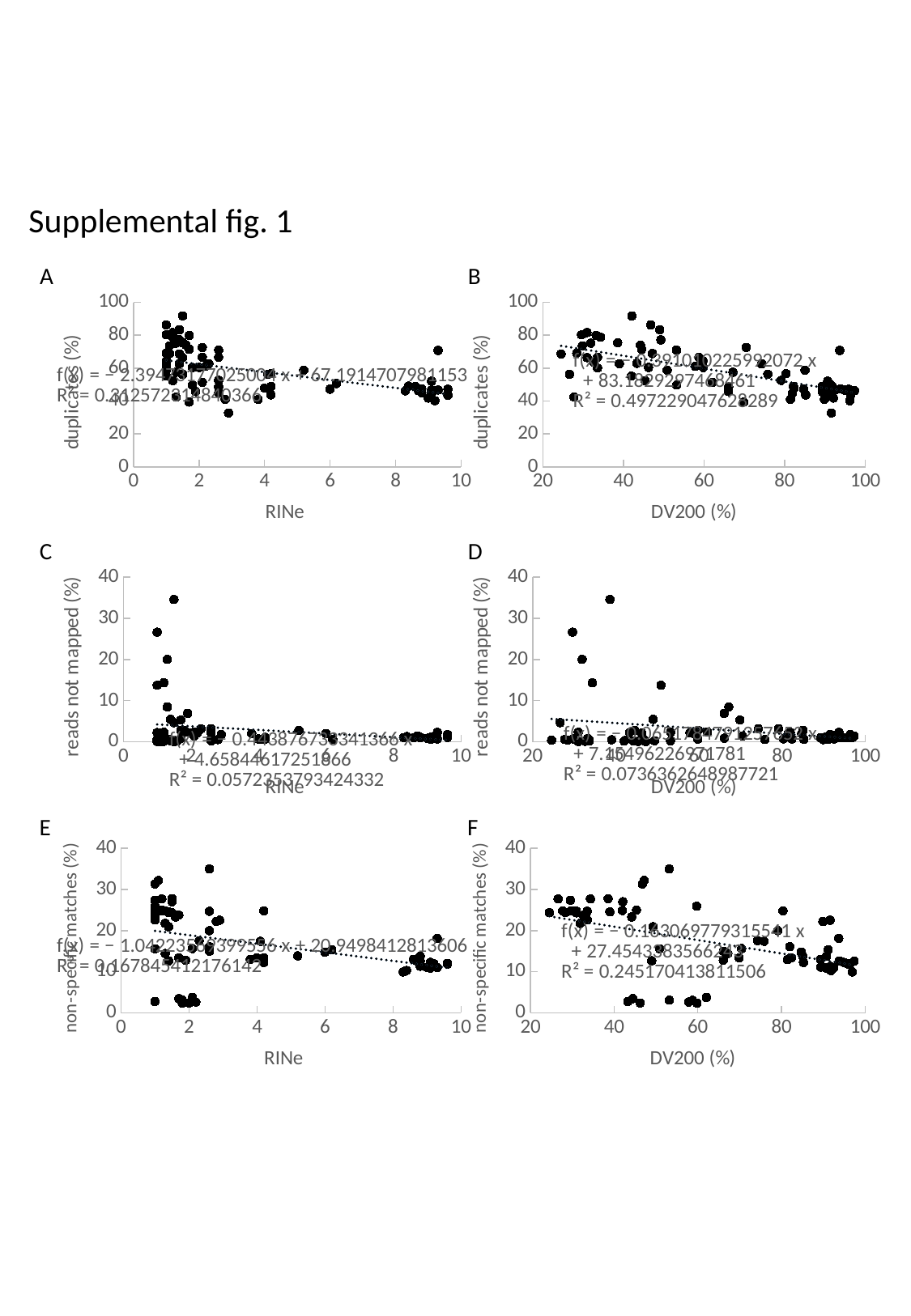

Supplemental fig. 1
A
B
### Chart
| Category | duplication rate(%) |
|---|---|
### Chart
| Category | |
|---|---|C
D
### Chart
| Category | not mapped reads (%) |
|---|---|
### Chart
| Category | not mapped reads (%) |
|---|---|E
F
### Chart
| Category | non-specific match |
|---|---|
### Chart
| Category | non-specific match |
|---|---|

## Slide 2
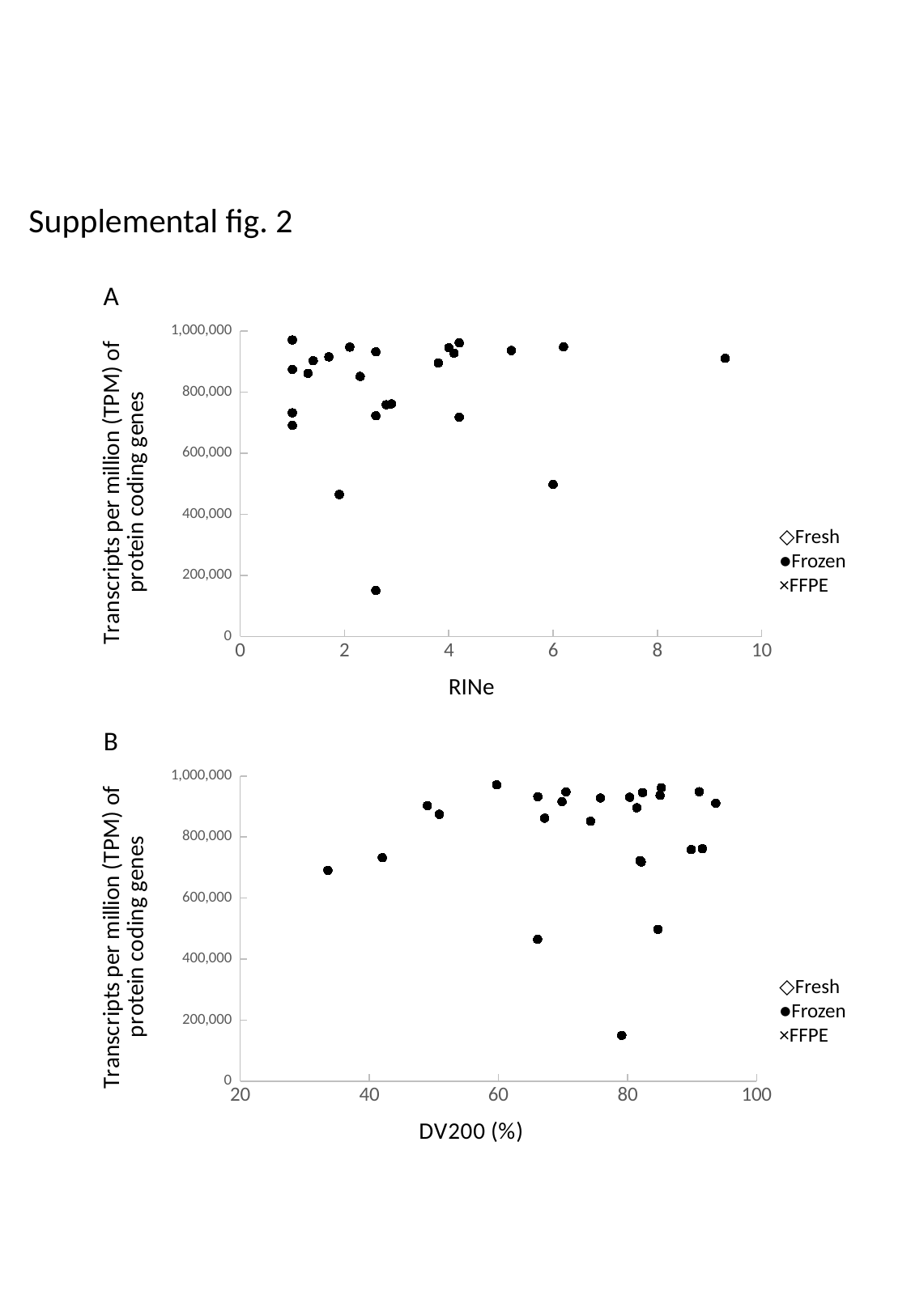

Supplemental fig. 2
A
### Chart
| Category | | | | |
|---|---|---|---|---|◇Fresh
●Frozen
×FFPE
B
### Chart
| Category | | | |
|---|---|---|---|◇Fresh
●Frozen
×FFPE
